# Aerosols and Bacteria From Hand Washing and Drying in Indoor Air

**DOI:** 10.3389/fpubh.2022.804825

**Published:** 2022-02-07

**Authors:** Maria Salomé Gião, Sotiris Vardoulakis

**Affiliations:** ^1^Dyson Technology Ltd., Malmesbury, United Kingdom; ^2^National Centre for Epidemiology and Population Health, Research School of Population Health, Australian National University, Canberra, ACT, Australia

**Keywords:** hand hygiene, washroom, aerosol, bacteria, jet air dryer, paper towel

## Abstract

Effective hand drying is an important part of hand hygiene that can reduce the risk of infectious disease transmission through cross-contamination of surfaces by wet hands. However, hand drying methods may also cause aerosolisation of pathogenic microorganisms if they are present in washed hands. This study investigated experimentally the impact of washing hands and different hand drying methods on the concentration and size distribution of aerosols and bacteria in indoor air. In this experiment, aerosol and bacteria concentrations were measured in indoor air while volunteers rinsed their hands with water or washed with soap and water prior to drying them with paper towels or jet air dryers. Results showed that the concentration of aerosols and bacteria in air increased with people walking in the room and washing hands, with a further increase during the hand drying process. The concentration of aerosols decreased with particle size, with maximum concentrations after drying hands of 6.63 × 10^6^ ± 6.49 × 10^5^ and 2.28 × 10^4^ ± 9.72 × 10^3^ particles m^−3^ for sizes 0.3 to <0.5 and ≥5.0 μm, respectively. The concentration of bacteria in indoor air after drying hands increased to a maximum of 3.81 × 10^2^ ± 1.48 × 10^2^ CFU m^−3^ (jet air dryers) and 4.50 × 10^2^ ± 4.35 × 10^1^ CFU m^−3^ (paper towels). This study indicates that the increase of aerosols and bacteria in air after drying hands with jet air dryers or paper towels are comparable and not statistically different from concentrations associated with walking and washing hands in the same environment. This work can support the development of hand hygiene practices and guidelines for public washrooms.

## Introduction

Human skin, in particular hands, harbors resident and transient microorganisms, which may include bacteria, viruses and fungi. Resident microorganisms are part of human skin flora, typically are not pathogenic and play a positive role in people's health (1, 2). Transient microorganisms are acquired due to human activities, such as handling raw food or visiting the toilet. Some transient microorganisms are innocuous to people, however others might be pathogenic and can transmit disease (1, 2). Effective hand washing can remove transient microorganisms, thus playing an important role in preventing disease transmission. Epidemiological studies have demonstrated that regular and adequate hand washing with soap and water can reduce the incidence of diarrhea, upper respiratory illness and other infectious diseases (3–5). As a result of the COVID-19 pandemic, there has been an increased focus on the importance of hand hygiene. Health organizations worldwide recommend frequent hand washing with soap and water followed by drying, or the use of disinfectant solutions, such as alcohol gels (6, 7).

Effective hand drying is increasingly recognized as an essential part of good hand hygiene (2, 8). It has been demonstrated that wet hands can transfer microorganisms to surfaces or become contaminated more readily (9–11). Public washrooms can offer several hand drying solutions, such as textile towels, paper towels and electric dryers. Textile and paper towels dry hands by absorbing water, while electric dryers dry hands through water evaporation by heated airflow (warm air dryers) or through removal of water by the action of high speed airflow (jet air dryers).

Several studies have shown that appropriate use of paper towels or jet air dryers decreases the number of bacteria in washed hands (12–14). However, it has been suggested that jet air dryers, due to their method of removing water, can create small aerosols that disperse microorganism in the washroom air. In fact, the hand drying process can generate aerosols, i.e., small solid or liquid particles suspended in the air (15), as well as larger ballistic droplets that settle gravitationally in seconds. Jet air dryers typically generate more ballistic droplets than paper towels. However, due to their larger size, these settle quickly on the floor or walls around the device (16) posing a relatively low risk of infection transmission (17). In terms of dispersion of microorganism in indoor air, the literature is inconsistent. A number of studies have shown comparable concentrations of bacteria in air for jet air dryers and paper towels (16, 18). Other studies have shown higher dispersal of bacteria and virus in the air when using electric dryers (19, 20), but in some cases their experimental methodologies employed gloved and unwashed hands, which were not representative of real world conditions (17). Furthermore, there is a lack of experimental studies that quantify the distribution of smaller aerosols (< 10 μm), which may remain suspended in air for long periods (minutes to hours), generated by different hand drying methods.

The objective of this work was to experimentally investigate the overall contribution of washing and drying hands with jet air dryers or paper towels to the concentration and distribution of small aerosols (0.3 μm to ≥ 5 μm), generated from the washing and drying process. The experiments were performed in a controlled environment to allow comparison of aerosol concentrations observed under different test conditions. The study hypothesis was that hand washing and drying methods significantly increase the concentration of aerosols in bathrooms. Additionally, the number of bacteria in air was measured to more holistically assess the impact of hand washing and drying methods on indoor air quality.

## Materials and Methods

### Experimental Set Up

**Figure 1:**
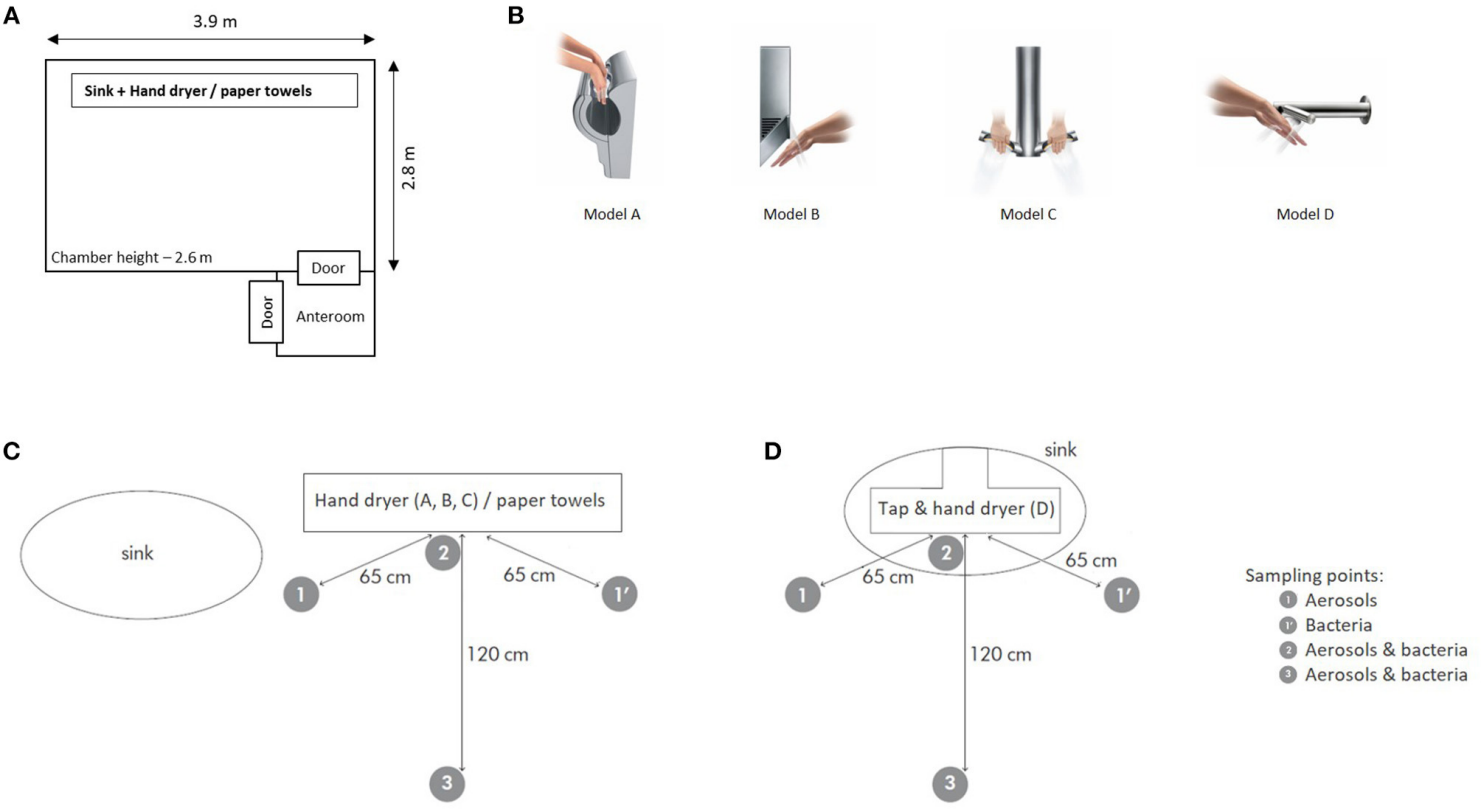
Diagrams representing **(A)** experimental chamber and **(B)** different models of jet hand dryers used in this study and hands position for drying. Sampling locations for **(C)** jet air dryer model A, B and C and paper towels and for **(D)** jet air dryer model D. Samplers are represented by the gray circle, with aerosol/particles counts samplers at locations 1, 2, and 3 and samplers for bacteria counts locations at 1', 2, and 3.

A 28.5 m^3^ chamber (Figure 1A) with environmental conditions controlled at 22°C and 55 % relative humidity, to represent typical washroom conditions (14), was used for the study. The ventilation was turned off during the course of the experiment and only volunteers were allowed in the chamber, in order to measure aerosolisation and bacteria counts due to hand drying activity. Before the start of the experiment the air was purged to remove contaminants (dust and other particles, bacteria) from the chamber air. To reflect their actual use, jet air dryer models A, B, and C (Figure 1B) were attached to a board placed vertically on the side of the sink, while model D was integrated with the tap on the sink. Paper towels were placed on a table near the jet air dryers models A, B, and C, folded in groups of 4 sheets to avoid cross-contamination between experiments.

**Figure 2:**
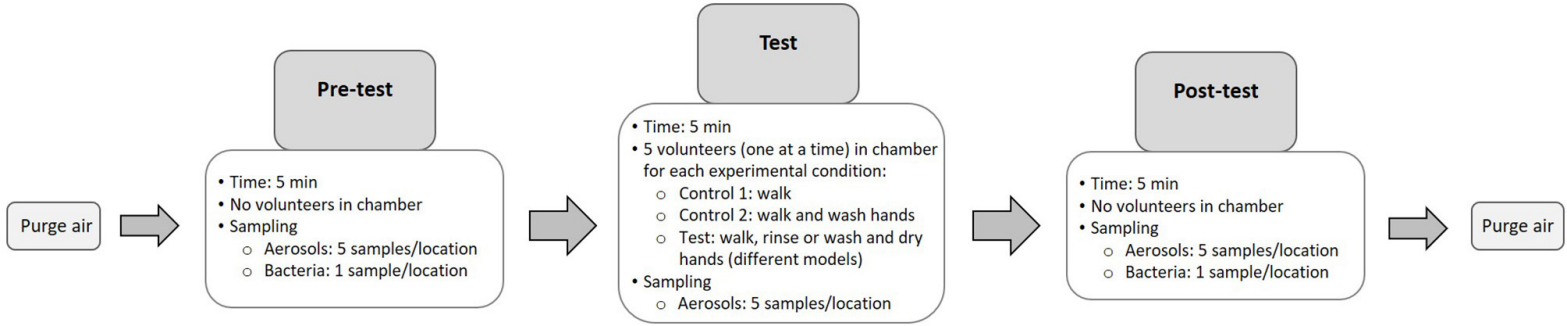
Diagram summarizing the experimental steps. Each experimental condition (from purge to post testing) were done in triplicate.

Figures 1C,D shows air samplers placed at three different locations in relation to the hand dryer, with particle counters placed at location 1, 2, and 3 to quantify aerosol number concentrations and bio-samplers placed at locations 1', 2, and 3 to quantify bacteria in air as total viable counts. The samplers were placed 1.5 m above the floor to represent the adult breathing zone (20). The air of the chamber was purged before the start of the experiment and pre-test (background) samples obtained to quantify aerosols and bacteria counts. Each volunteer entered the room, walked to the sink, rinsed or washed hands, walked to the side to jet air dryer models A, B and C and paper towels, or remained at the sink for model D, and left the room. This was repeated sequentially until 5 volunteers performed the same procedure (Table 1). After volunteer 5 left the chamber, the air was sampled for 5 min and then purged (Figure 2). The experiment was repeated 2 more times for each condition (control experiment and hand drying method) with different groups of volunteers. The triplicates for each experiment were done sequentially before moving to the next experimental condition.

**Table 1:** Description of experimental conditions and sample size for control and test (use of different hand drying methods) experiments.

**Experimental condition**	**Description of experimental activities**	**Sample size**
Control 1 - walk	The volunteer entered the chamber, walked to the sink and waited 20 s. The volunteer then waited 10 s next to the hand dryer and exited the chamber	
Control 2 – walk and wash	The volunteer entered the chamber, walked to the sink and washed hands with soap for 20 s. The volunteer then waited 10 s next to the hand dryer and exited the chamber	5 volunteers: volunteer 1 entered, performed experimental procedure and exited. Volunteer 2 entered and repeated
Test - Rinse and dry hands	The volunteer entered the chamber, walked to the sink and rinsed hands (with only water) for 20 s. The volunteer then dried hands using one of the hand drying methods for the time stated on Table 2 and exited the chamber	procedure of volunteer 1. This was repeated until 5 volunteers performed the same experimental procedure
Test - Wash and dry hands	The volunteer entered the chamber, walked to the sink and washed hands with soap for 20 s. The volunteer then dried hands using one of the hand drying methods for the time stated on Table 2 and exited the chamber	

*Each experimental condition was performed in triplicate with different volunteers, with a total of 15 volunteers for each control or test condition*.

To characterize the impact of walking and washing hands on aerosol and bacteria concentrations in the air, two control experiments were performed. In control experiment 1, the volunteer walked to the sink and waited 20 s without activating the tap, then walked to the dryer and waited 10 s without using the dryer or paper towel before leaving the chamber. In control experiment 2, the volunteer followed the same procedure, but also washed hands with water and soap for 20 s.

### Hand Washing and Drying

The experiments were performed in a controlled environment with a total of 15 volunteers for each control or test condition. The volunteers were asked to wear coverall suits (Chemsplash, UK) that were cleaned with 70% ethanol prior to each experiment. For each hand drying method two different experimental test conditions were performed. On a first test condition volunteers rinsed their hands for 20 s in water without using any soap in order to represent poorly washed hands. In a second test condition, volunteers washed their hands with soap and water for 20 s following WHO guidelines (8). After rinsing or washing hands, volunteers dried them up with a jet air dryer or paper towels. When using jet air dryers, volunteers dried their hands for the time recommended by the manufacturer (10–14 s, depending on the model), while when using paper towels volunteers dried their hands until they felt completely dry (average drying time measured: 6.1 ± 1.4 s).

Four different models of jet air dryers (Dyson Technology Ltd, UK) were used in this experiment, which represent a range of jet air dryers commonly found in public washrooms. All four models had high efficiency particulate air (HEPA) filters fitted, but different design and air outlet configuration (Figure 1B), drying time (10–14 s), airflow (20–30 L s^−1^), and air velocity (152–192 m s^−1^), as summarized in Table 2. In jet air dryer model A, hands were inserted from the top and moved up and down four times. When using jet air dryer models B and C, hands were placed under the hand dryer and moved toward the volunteer and back four times, changing hand side each time. In model B, air exits through straight apertures, while model C has curved air apertures. Model D is an integrated dryer on a tap; hands were washed and immediately dried on the sink by placing them under the dryer's arms and moved toward the volunteer and back four times, changing hand side each time. To dry hands with paper towels (Professional Hygiene Ltd., UK) 4 sheets of paper were used each time. The number of paper sheets needed was determined in a preliminary laboratory experiment, where volunteers used different numbers of sheets to dry hands completely.

**Table 2:** Values for recommended drying time, airflow rate and air velocity (at the outlet) for the different jet air dryer models (values provided by the manufacturer).

**Hand dryer model**	**Position of hands in relation to dryer and type of drying**	**Airflow rate (L s^**−1**^)**	**Air velocity (m s^**−1**^)**	**Dry time (s)**
A	Hands in, dry both sides of hands simultaneously	30	192	10
B	Hands under, dries one side of the hands separately	20	192	12
C	Hands under, dries one side of the hands separately	23	173	10
D	Hands under, dries one side of the hands separately, hand dryer on the sink.	21	152	14

### Bacteria in Air and Aerosol Sampling

The concentration and size distribution of aerosols was measured using a particle counter CLiMET CI-450 (at locations 1 and 2) and CI-754 (modified by the manufacturer to measure a wider range of particles size, location 3) (CLiMET Instruments, USA). These samplers were used to measure the concentration of aerosols of different sizes every minute for a total of 15 min for every group of volunteers: 5 time points before the first volunteer entered the chamber (pre-test) to quantify background aerosols, 5 time points while volunteers were in the chamber (test) and 5 time points after the 5^th^ volunteer left the chamber (post-test). The size of the particles measured ranged between 0.3 and ≥5 μm, divided into 4 bins, representing different size ranges as defined in Table 3. Location 3 had different bin sizes to measure larger aerosols.

**Table 3:** Aerosols size range for each bin for each location.

**Location**	**Aerosol parameters**				
1 & 2	Bin	0.3	0.5	1.0	5.0
	Size (μm)	0.3 to <0.5	0.5 to <1.0	1.0 to <5.0	≥5.0
3	Bin	0.3	1.0	3.0	10
	Size (μm)	0.3 to <1.0	1.0 to <3.0	3.0 to <10.0	≥10

The number of bacteria in air was measured as total viable count using Biostage impactors (SKC BioStage® 400-hole viable cascade impactor, SKC, USA), collecting air by impaction on tryptic soy agar (TSA, BIOKAR Diagnostics, France) plates. For each experiment, one sample at each location was collected before the first volunteer entered the room (pre-test) and after the 5^th^ volunteer exited (post-test). Each sample was collected at a flow rate of 21 L min^−1^ for 5 min; the bio-sampler was calibrated to this flow rate to prevent potential overgrowth on agar plates. The TSA plates were incubated at 37°C for 48 h before colony forming units (CFU) were counted.

### Statistical Analysis

In order to account for the inherent variability in background aerosol and bacteria concentrations in air before each experiment, the data were normalized. Aerosol numbers were normalizing by dividing each data point by the average of the 5 first data points corresponding to concentration of aerosols during the pre-test (i.e., measurements carried out in the clean chamber). Bacteria numbers were presented in a logarithmic (base 10) scale. The statistical analysis was performed using GraphPad Prism for Windows (version 9.0.0 GraphaPad Software, USA). Statistically significant differences were calculated using two-way analysis of variance (ANOVA) and Tukey tests for multiple comparisons. The increase was statistically significant for values of *p* < 0.05.

## Results

### Background Concentration of Aerosols and Bacteria

The background concentration of aerosols (pre-test values) differed according to the particle size measured, with a decrease of concentration as the size increased. The background concentration of aerosols ranged between 4.92 × 10^5^ ± 3.00 × 10^4^ and 1.43 × 10^6^ ± 2.63 × 10^5^ particles m^−3^ for aerosols in bin 0.3, and between 1.79 × 10^2^ ± 6.84 × 10^1^ and 2.81 × 10^3^ ± 1.96 × 10^2^ particles m^−3^ for aerosols bins 5.0 and 10.0, respectively. The background concentration of bacteria varied between 9.63 ± 0.00 and 1.21 × 10^2^ ± 4.90 × 10^1^ CFU m^−3^.

### Contribution of Non-drying Activities to Aerosols and Bacteria Counts (Controls)

**Figure 3:**
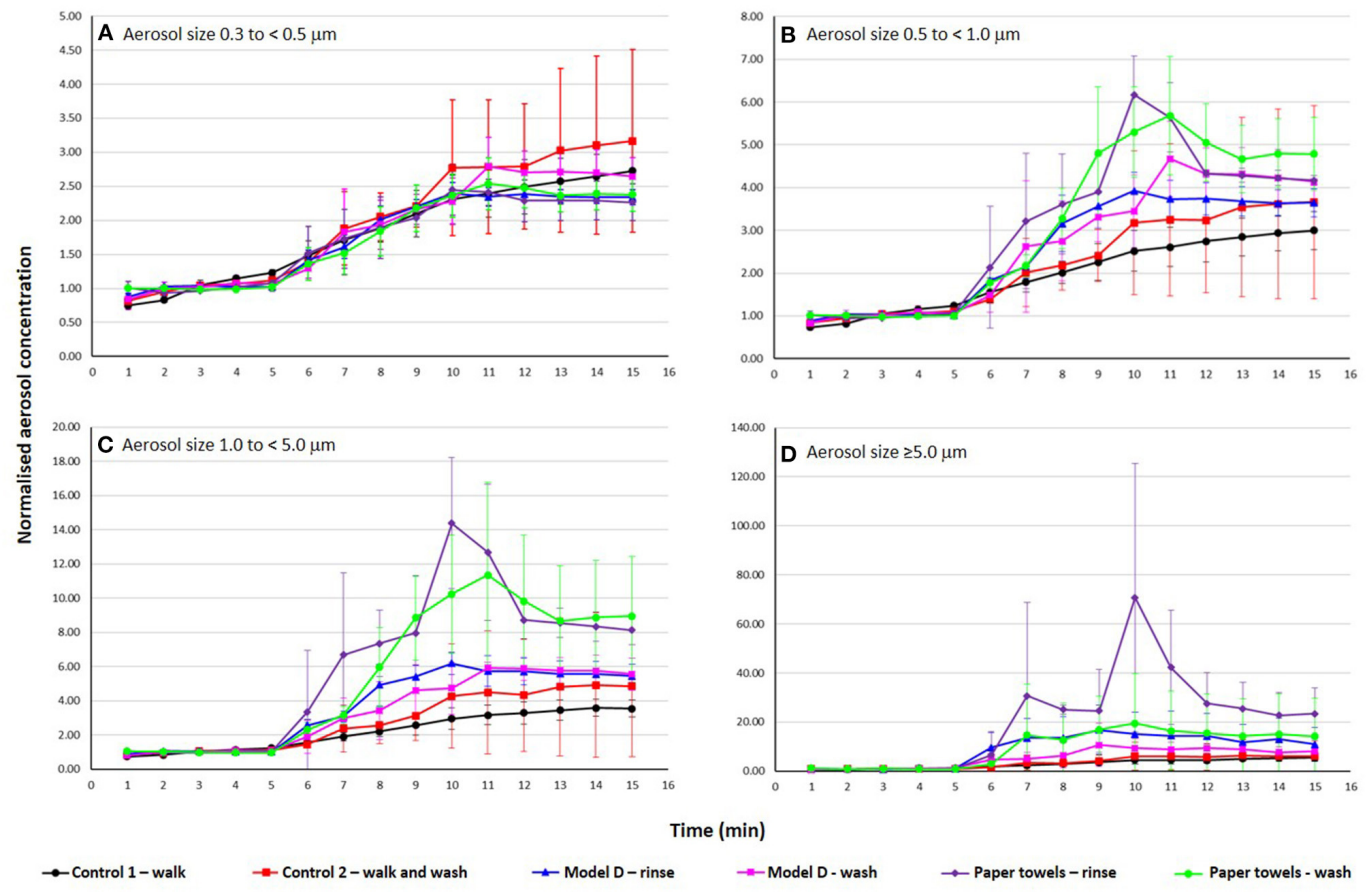
Normalized concentrations for model D for aerosol bins **(A)** 0.3, **(B)** 0.5, **(C)** 1.0, and **(D)** 5.0 for location 2 (near the hand dryer). Each curve represents (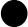) control experiment 1 (walking only), (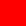) control experiment 2 (walking and hand washing), and drying hands with jet air dryer model D after (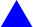) rinsing and (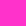) washing, and drying with paper towels after (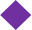) rinsing and (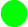) washing. Vertical bars represent standard deviation of triplicates.

**Figure 4:**
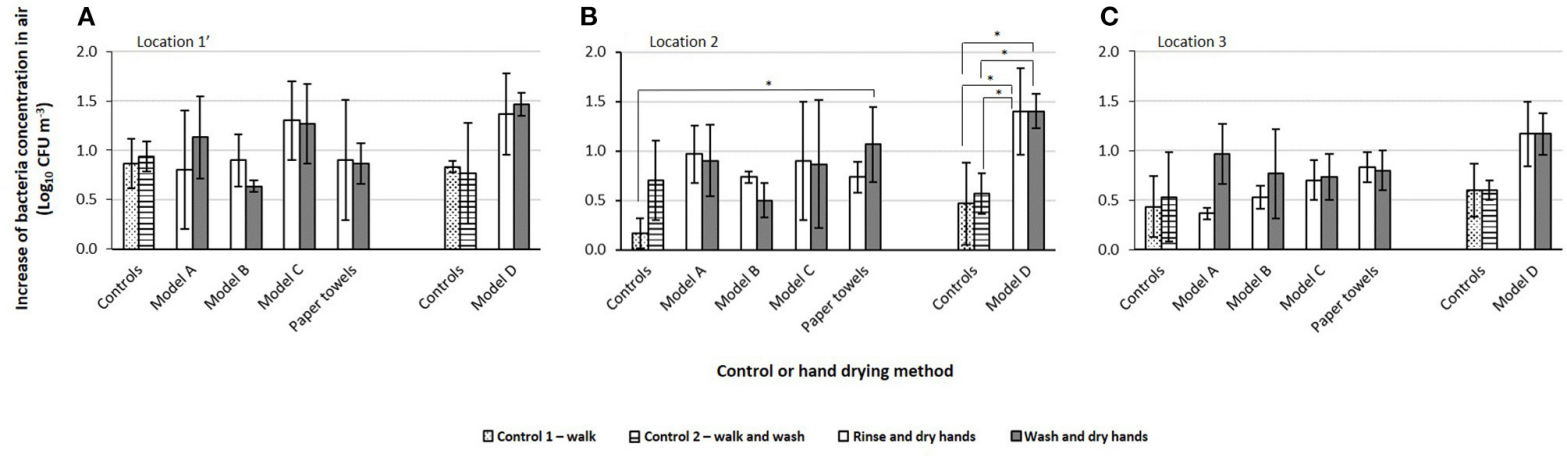
Increases in bacteria concentrations at each sampling location: **(A)** 1', side of the dryer, **(B)** 2, near the dryer, and **(C)** 3, opposite the dryer, for controls and for each hand drying method. Bars with patterns represent controls experiment 1 and 2: walking only (bars with dots) and walking and washing (bars with stripes); bars with solid fill represent tests, with drying after rinsing hands (white bars) and after washing with soap (gray bars). Asterisks indicate comparisons in which differences are statistically significant (*p* < 0.05). Vertical bars represent the standard deviation of triplicates.

Concentration of aerosols and bacteria were measured when volunteers only walked in the chamber and when volunteers walked and washed hands, but did not dry them (control experiments 1 and 2). The results obtained for aerosols showed a slightly higher increase when volunteers walk and wash hands compared to only walking (Figure 3 and Supplementary Figure 1.1–1.11) for all particles size and locations, however the results from control experiments 1 and 2 were not statistically different (*p* > 0.05). Figure 4 shows the increase in bacteria concentration in air for control experiments 1 and 2. The difference between control 1 (walk) and control 2 (walk and wash) was not statistically different (*p* > 0.05). The concentrations of bacteria in air were on average 8.00 × 10^1^ ± 2.73 × 10^1^ and 5.82 × 10^1^ ± 1.30 × 10^1^ CFU m^−3^ after the volunteers walked in and out of the chamber, and 1.01 × 10^2^ ± 8.64 and 1.44 × 10^2^ ± 3.82 × 10^1^ CFU m^−3^ after walking into, washing hands and leaving the chamber, for controls with samplers positioned on the side of the sink (Figure 1C) and above the sink (Figure 1D), respectively.

### Impact of Hand Drying on Aerosolisation

To study the effect of hand drying on aerosolisation, the concentration and size distribution. The maximum concentration of aerosols sampled during experiments when a volunteer dried hands with a jet air dryer was 6.63 × 10^6^ ± 6.49 × 10^5^ (model A) and 1.05 × 10^4^ ± 7.33 x10^2^ (model B) particles m^−3^, for bins 0.3 and ≥5.0, respectively. When volunteers used paper towels to dry hands the maximum concentration of aerosols measured was 3.49 × 10^6^ ± 4.95 × 10^5^ and 2.28 × 10^4^ ± 9.72 × 10^3^ particles per m^3^, for bins 0.3 and ≥5.0, respectively. Supplementary Figure 2.1–2.3 show the average concentration of aerosols for the different hand drying methods for pre-test, test and post-test samples collected at each sampling location. Although the air was purged before all experiments, there were background aerosols present in the air before the first volunteer from each group entered the chamber, which are different for each experimental run. This variation resulted in inaccurate comparisons of aerosols concentration for the different experiments. Therefore, in order to compare results, it was necessary to normalize the data by calculating the increase of aerosols in relation to the background. Figure 3 shows the normalized concentration (increase) of aerosols over time obtained for controls, jet air dryer model D and paper towels at location 2. At this location, controls, drying hands with jet dryer model D or paper towels, had similar increase of the concentration of the smallest (bin 0.3 μm) aerosols (*p* > 0.05). Drying hands with jet air dryer model D, both after rinsing and washing, resulted in a lower increase of aerosols in bins 0.5, 1.0, and 5.0 μm compared to drying hands with paper towels, but differences were not statistically significant (*p* > 0.05). There was also a slight increase in aerosol concentrations when the volunteers dried hands with jet dryer model D compared to washing but not drying (control experiment 2), which was not statistically significant (*p* > 0.05). The results for the other sampling locations follow a similar pattern (Supplementary Figure 1.4, 1.11).

For the experiments with jet air dryer models B (Supplementary Figure 1.2, 1.6, 1.9) and C (Supplementary Figure 1.3, 1.7, 1.10) results showed a similar pattern to model D, the increase of aerosol concentrations across sizes and locations is lower compared to paper towels, but in general not statistically significant (*p* > 0.05). Comparing to walking and washing hands, the increase seen for jet dryers is not statistically significant (*p* > 0.05). For model A (Supplementary Figure 1.1, 1.5, 1.8) smaller aerosols sizes (bins 0.3 and 0.5) had a higher increase in concentrations compared to paper towels, but the difference was not significant (*p* > 0.05).

Supplementary Figure 1.1–1.11 shows the increase in aerosol concentrations across all settings, which was more pronounced for larger particle sizes. There was no relationship between different levels of increase and sampling location (horizontal distance to the dryer).

### Impact of Hand Drying on Bacteria in Air

To determine the influence of hand drying on bacteria concentrations in air, volunteers either rinsed or washed hands prior to drying them with one of the jet air dryer models or paper towels. The final concentration of bacteria in air after drying hands with jet air dryers varied between a minimum of 1.30 × 10^2^ ± 3.49 × 10^1^ CFU m^−3^ and a maximum of 3.81 × 10^2^ ± 1.48 × 10^2^ CFU m^−3^ when jet air dryer model C and D were used, respectively. When hands were dried up with paper towels, the concentration of bacteria in air varied between 9.08 × 10^1^ ± 2.05 × 10^1^ and 4.50 × 10^2^ ± 4.35 × 10^1^ CFU m^−3^.

Figure 4 shows the increase in bacteria concentrations in air after drying hands with different methods, for the three sampling locations. Hand drying promoted an increase in bacteria concentrations for both drying methods, i.e., jet air dryers and paper towels. The extent of the increase depended on the drying method used, with model B accounting for the smallest increase, on average 0.68 ± 0.15 log_10_ CFU m^−3^. There was no statistically significant difference in the increase of bacteria concentrations in air after drying hands with jet air dryer models A, B and C compared to walking and washing hands or drying them with paper towels at any of the locations sampled (*p* > 0.05). Model D showed the highest increase in bacteria concentrations in air, on average 1.33 ± 0.13 log_10_ CFU m^−3^, but this increase was not statistically different compared to paper towels (*p* > 0.05). The increase of bacteria after drying hands with paper towels was on average 0.87 ± 0.13 log_10_ CFU m^−3^, however the difference was statistically different only when drying after rinsing was compared to walking at location 2 (*p* = 0.02). Comparing the increase in bacteria concentrations between model D and control experiments 1 (walking) and 2 (walking and washing hands), statistically significant increases were observed (*p* = 0.01 for control 1 and *p* = 0.04 for control 2) for both rinsing and washing with soap experiments for samples obtained near the hand dryer and sink (location 2). However, these increases were not statistically significant for locations 1' and 3 (*p* > 0.05).

### Comparison of Bacterial and Aerosols Increase When Hands Are Rinsed or Washed

To evaluate any differences in bacteria concentrations in air and aerosols when people did not comply with appropriate hand washing, volunteers rinsed hands with only water or washed them with soap and water for 20 s, prior to drying them. Results for both aerosol (all sizes) and bacteria number concentrations showed no statistical difference between rinsing and washing (*p* > 0.05) for all jet air dryer models and paper towels at all locations.

## Discussion

Aerosols and bacteria are ubiquitous in bathrooms (21). Several human activities can contribute to their increase indoors, including walking, changing clothes, and flushing the toilet (22, 23). The concentrations of aerosols of different size ranges, from 0.3 to ≥ 5 μm, in the chamber experiments showed an increase in aerosols concentration when people walked, washed and dried hands. The increase of aerosols of all sizes generated from drying hands with paper towels was not statistically significant compared to the increase generated by drying hands with jet air dryer models B, C, and D. However, there was a pattern indicating that drying hands with paper towels generates more aerosols than when using these jet air dryer models. Aerosols generated by jet air dryers are likely to be liquid aerosols, while aerosols from drying hands with paper towels may contain dust from the paper, which could explain the pattern of a higher aerosol concentration increase when volunteers dried hands with paper towels, in particular for bigger aerosols. Jet air dryer model A showed higher increase of aerosols compared to paper towels for smaller aerosols (bin 0.3). Although the increase was not statistically different, it shows a different pattern compared to the other jet air dryer models. This could be explained by the higher airflow or different configuration of this model (Figure 1B and Table 2), i.e., air is blown upwards for model A while for models B, C, and D air is blown downwards. It was also observed for location 1 (Supplementary Figure 1.1–1.4) that the increase of aerosols when hands were dried with paper towels after being rinsed is lower than when hands are dried with paper towels after being washed or after being dried with one of the jet hand dryers. While no statistical difference was found between these results (*p* > 0.05), it is possible this difference is due to the initial concentration of aerosols (Supplementary Figure 2.1) or influence of not using soap during hand rinsing. It would be beneficial to study this further to understand the potential impact of different hand washing methods on aerosolisation during hand drying.

The results presented here show that there is a relatively small contribution from drying hands with jet air dryers to the aerosol concentrations in the air. This is consistent with previous studies showing that the water is mostly removed in the form of ballistic droplets that fall on the floor and walls close to the hand drying unit (16, 19).

The concentration of aerosols decreased with particle size before the volunteers entered the room. This is consistent with a study on aerosols in washrooms by Knowlton et al., which showed decreased number concentrations with aerosol size (22). As volunteers entered the chamber and left again, the number of aerosols increased and stabilized after the last volunteer left. The smaller the aerosol size measured the smaller the increase, showing that a higher proportion of larger aerosols was created (Figure 3). Knowlton et al. measured higher increases in smaller aerosols when the toilet was flushed (22).

There are no international standards that specify limits for bacteria in indoor air, but national or regional guidelines stipulate levels that should not be exceeded, which vary from 5.00 × 10^2^ to 1.00 × 10^4^ CFU m^−3^ (24). The results in this study show that walking and washing hands increased the number of bacteria in indoor air compared to just walking. Drying hands with paper towels or jet air dryers increased those concentrations in air further, but the increases were not significantly different to walking or washing hands without drying them up. The exception was for drying hands with paper towels (after rinsing) compared to walking, and for drying hands with jet air dryer model D (after rinsing and washing) compared to walking or walking and washing hands, which were statistical significant at location 2, possibly because this sampling location was the nearest to the hand drying location. The highest increase in the number of bacteria was for model D, an expected result since this device was placed on the sink, which was wet and may have contained bacteria; however the number of bacteria in air were below acceptable levels, according to regional guidelines for bacteria in indoor air reported by Guo et al. (24). For all jet air dryer models tested, the increase of bacteria in air was not statistically different compared to drying hands with paper towels and to washing without drying up hands. This indicates that drying hands with jet air dryers has no significant influence on the bacterial load in air, possibly because the majority of the water is removed in the form of large droplets that fall on the floor and walls (16, 19).

The non-statistically significant impact of jet air dryers on bacterial load in air is consistent with results from other studies, which also obtained comparable numbers when hands were dried with jet air dryers or paper towels (16, 18). Other authors have reported that drying hands with jet air dryers resulted in much higher level of virus and bacteria concentrations in air (19, 20), however these experiments employed unrealistic experimental conditions (17, 21).

Furthermore, this work indicates that there was no significant difference in the increase of aerosols and bacteria concentrations in air if hands were just rinsed instead of washed with soap and water.

## Limitations of the Study

The experimental work in this study was done in a clean chamber with no other activities performed simultaneously, in order to isolate the contribution of hand drying to aerosol and bacterial loads in indoor air. In a real washroom, other activities would also be contributing to these loads, and some washrooms also have different hand drying methods available simultaneously. It is suggested that future studies measure concentrations of aerosols and bacteria in air when more than one drying method is available and also perform experiments in real washrooms. The smallest aerosol size measured in this study was 0.3 μm due to the equipment detection limit; however this is approximately the size of the smallest bacteria. In this work, the concentration of bacteria in air was measured, but no identification of the species was done. This could have given important information about the presence of potential pathogens, and it is therefore recommended to include this analysis in future experiments.

## Conclusions

Appropriate hand hygiene requires thorough hand washing to remove dirt and microorganisms, followed by effective hand drying. It is important that hand drying methods do not re-contaminate washed hands or contaminate washroom air from aerosolisation of particles or bacteria. The results presented in this work showed that there is no significant difference in the increase of aerosol or bacteria concentrations in indoor air when people wash hands compared to just walking. Furthermore, there is no significant difference in the increase of aerosol or bacteria concentrations for the two hand drying methods employed, jet air dryers or paper towels. Although hand drying can increase aerosol and bacteria concentrations in indoor air, the difference between only washing hands compared to washing and drying hands was not statistically significant in our study.

This work indicates that both hand drying methods, i.e., electric hand dryers and paper towels, have a relatively small impact on aerosols and bacteria concentrations in indoor air compared to other common activities, such as walking and washing hands. Our findings can inform the development of hand hygiene policies and guidelines for the safe use of public washrooms. Further research is needed to holistically assess hand drying methods in different indoor settings under a variety of environmental conditions and ventilation regimes.

## Data Availability Statement

The raw data supporting the conclusions of this article will be made available by the authors, without undue reservation.

## Author Contributions

MSG: conceptualization, experimental design and data analysis. MSG and SV: writing, review, and editing. All authors contributed to the article and approved the submitted version.

## Funding

The experimental work was financially supported by Dyson Technology Ltd. (UK).

## Conflict of Interest

Author MSG was an employee of Dyson. Author SV was member of the Dyson Scientific Advisory Board and had received research funding and honoraria from Dyson. This study received funding from Dyson Technology Ltd. (UK). The funder had involvement in the conceptualization of the study.

## Publisher's Note

All claims expressed in this article are solely those of the authors and do not necessarily represent those of their affiliated organizations, or those of the publisher, the editors and the reviewers. Any product that may be evaluated in this article, or claim that may be made by its manufacturer, is not guaranteed or endorsed by the publisher.

## References

[1] Byrd AL Belkaid Y Segre JA. The human skin microbiome. Nat Rev Microbiol. (2018) 16:143–55. 10.1038/nrmicro.2017.157 29332945

[2] Vandegrift R Bateman AC Siemens KN Nguyen M Wilson HE Green JL. Cleanliness in context: reconciling hygiene with a modern microbial perspective. Microbiome. (2017) 5:76. 10.1186/s40168-017-0294-2 28705228 PMC5513348

[3] Aiello AE Larson EL. What is the evidence for a causal link between hygiene and infections? Lancet Infect Dis. (2002) 2:103–10. 10.1016/S1473-3099(02)00184-6 11901641

[4] Black RE Dykes AC Anderson KE Wells JG Sinclair SP Gary GW. Handwashing to prevent diarrhea in day-care centers. Am J Epidemiol. (1981) 113:445–51. 10.1093/oxfordjournals.aje.a113112 7211827

[5] Rabie T Curtis V. Handwashing and risk of respiratory infections: a quantitative systematic review. Trop Med Int Health. (2006) 11:258–67. 10.1111/j.1365-3156.2006.01568.x 16553905 PMC7169664

[6] CDC. COVID-19: How to Protect Yourself and Others. (2020). Available online at: https://www.cdc.gov/coronavirus/2019-ncov/prevent-getting-sick/prevention.html?CDC_AA_refVal=https%3A%2F%2F2019-ncov%2Fprepare%2Fprevention.html (accessed January 13, 2021).

[7] WHO. Clean Hands Protect Against Infection. (2020). Available online at: https://www.who.int/teams/integrated-health-services/infection-prevention-control (accessed October 29, 2020).

[8] WHO. WHO Guidelines on Hand Hygiene in Health Care. Geneva: World Health Organization (2009).

[9] Lopez GU Gerba CP Tamimi AH Kitajima M Maxwell SL Rose JB. Transfer efficiency of bacteria and viruses from porous and nonporous fomites to fingers under different relative humidity conditions. Appl Environ Microbiol. (2013) 79:5728–34. 10.1128/AEM.01030-13 23851098 PMC3754157

[10] Patrick DR Findon G Miller TE. Residual moisture determines the level of touch-contact-associated bacterial transfer following hand washing. Epidemiol Infect. (1997) 119:319–25. 10.1017/S0950268897008261 9440435 PMC2809004

[11] Sattar SA Springthorpe S Mani S Gallant M Nair RC Scott E. Transfer of bacteria from fabrics to hands and other fabrics: development and application of a quantitative method using Staphylococcus aureus as a model. J Appl Microbiol. (2001) 90:962–70. 10.1046/j.1365-2672.2001.01347.x 11412326

[12] Mutters R Warnes SL. The method used to dry washed hands affects the number and type of transient and residential bacteria remaining on the skin. J Hosp Infect. (2019) 101:408–13. 10.1016/j.jhin.2018.12.005 30537524

[13] Snelling AM Saville T Stevens D Beggs CB. Comparative evaluation of the hygienic efficacy of an ultra-rapid hand dryer vs conventional warm air hand dryers. J Appl Microbiol. (2011) 110:19–26. 10.1111/j.1365-2672.2010.04838.x 20887403 PMC3017747

[14] Suen LKP Lung VYT Boost MV Au-Yeung CH Siu GKH. Microbiological evaluation of different hand drying methods for removing bacteria from washed hands. Sci Rep. (2019) 9:13754. 10.1038/s41598-019-50239-4 31551459 PMC6760209

[15] Kulkarni P Baron PA Willeke K. Introduction to Aerosol Characterization, in: Aerosol Measurement. John Wiley & Sons, Ltd., (2011). 10.1002/9781118001684.ch125855820

[16] Margas E Maguire E Berland CR Welander F Holah JT. Assessment of the environmental microbiological cross contamination following hand drying with paper hand towels or an air blade dryer. J. Appl. Microbiol. (2013) 115:572–82. 10.1111/jam.12248 23683001

[17] Reynolds KA Sexton JD Norman A McClelland DJ. Comparison of electric hand dryers and paper towels for hand hygiene: a critical review of the literature. J Appl Microbiol. (2021) 130:25–39. 10.1111/jam.14796 32794646 PMC7818469

[18] Best E Parnell P Couturier J Barbut F Le Bozec A Arnoldo L. Environmental contamination by bacteria in hospital washrooms according to hand-drying method: a multi-centre study. J Hosp Infect. (2018) 100:469–75. 10.1016/j.jhin.2018.07.002 30006281

[19] Best EL Parnell P Wilcox MH. Microbiological comparison of hand-drying methods: the potential for contamination of the environment, user, and bystander. J Hosp Infect. (2014) 88:199–206. 10.1016/j.jhin.2014.08.002 25237036

[20] Kimmitt PT Redway KF. Evaluation of the potential for virus dispersal during hand drying: a comparison of three methods. J Appl Microbiol. (2016) 120:478–86. 10.1111/jam.13014 26618932

[21] Vardoulakis S Espinoza Oyarce DA Donner E. Transmission of COVID-19 and other infectious diseases in public washrooms: A systematic review. Sci Total Environ. (2022) 803:149932. 10.1016/j.scitotenv.2021.149932 34525681 PMC8390098

[22] Knowlton SD Boles CL Perencevich EN Diekema DJ Nonnenmann MW. Bioaerosol concentrations generated from toilet flushing in a hospital-based patient care setting. Antimicrob Resist Infect Control. (2018) 7:16. 10.1186/s13756-018-0301-9 29423191 PMC5787296

[23] Luoma M Batterman SA. Characterization of particulate emissions from occupant activities in offices. Indoor Air. (2001) 11:35–48. 10.1034/j.1600-0668.2001.011001035.x 11235230

[24] Guo K Qian H Zhao D Ye J Zhang Y Kan H. Indoor exposure levels of bacteria and fungi in residences, schools, and offices in China: A systematic review. Indoor Air. (2020) 30:1147–65. 10.1111/ina.12734 32845998

