# Supplementary material for: Aerosols and Bacteria From Hand Washing and Drying in Indoor Air
Source: Front Public Health. 2022 Feb 7;10:804825. doi: 10.3389/fpubh.2022.804825 (PMC8858938; doi:10.3389/fpubh.2022.804825)
Supplement: Supplementary file 1 [file Data_Sheet_1.PDF]

Supporting information Giao and Vardoulakis – Graphs for aerosols measured at all locations and for all jet dryers and paper towels.

S1 – Normalised aerosol concentration with time

Location 1

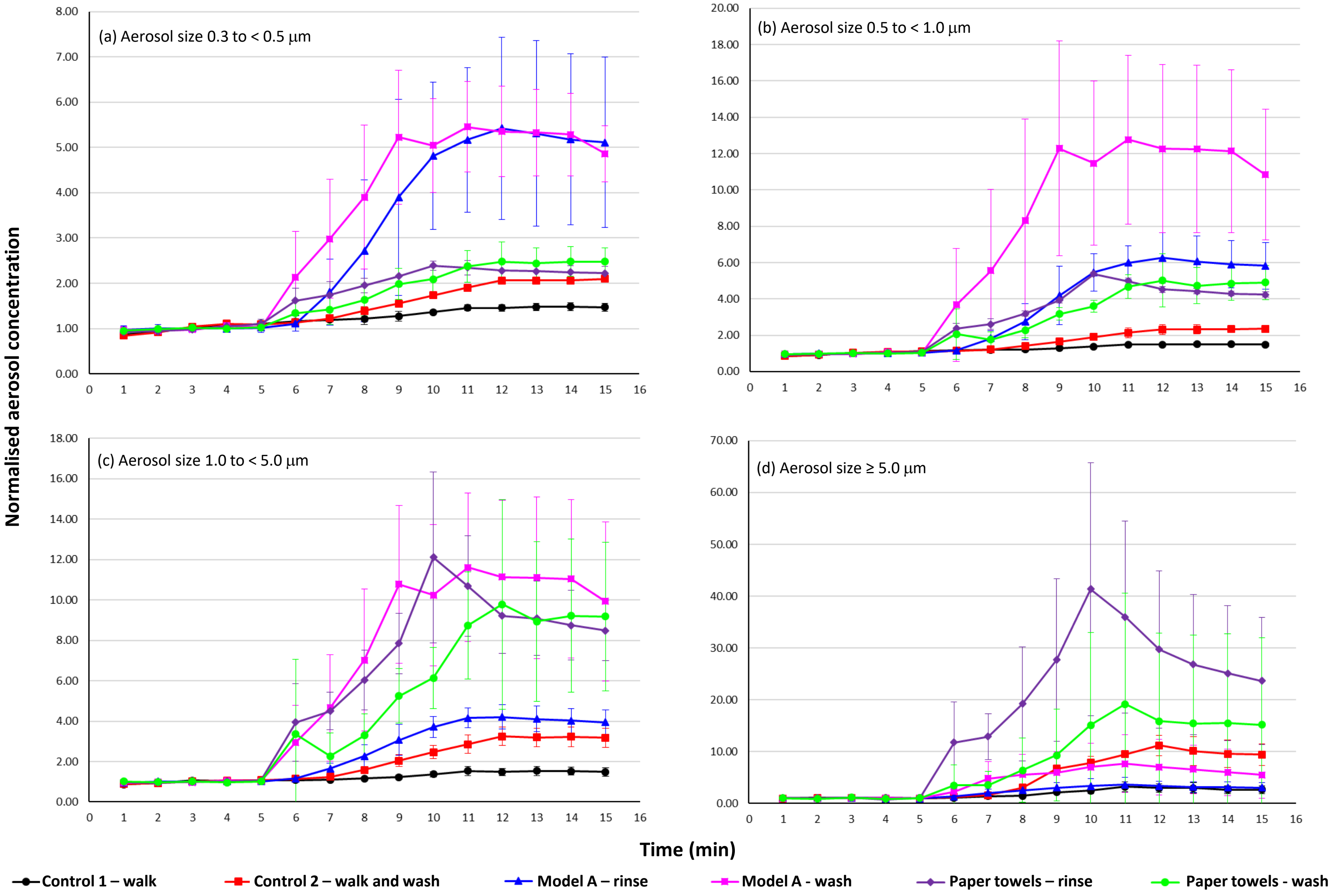

Figure S1.1. Representation of normalised data for model A for aerosol bins a) 0.3, b) 0.5, c) 1.0 and d) 5.0 for location 1 (side of hand dryer). Each curve represents (●) control 1 (walking only), (■) control 2 (walking and hand washing), and drying hands with jet dryer model A after (▲) rinsing and (■) washing, and drying with paper towels after (◆) rinsing and (●) washing. Vertical bars represent standard deviation of three experiments.

S1 – Normalised aerosol concentration with time

Location 1

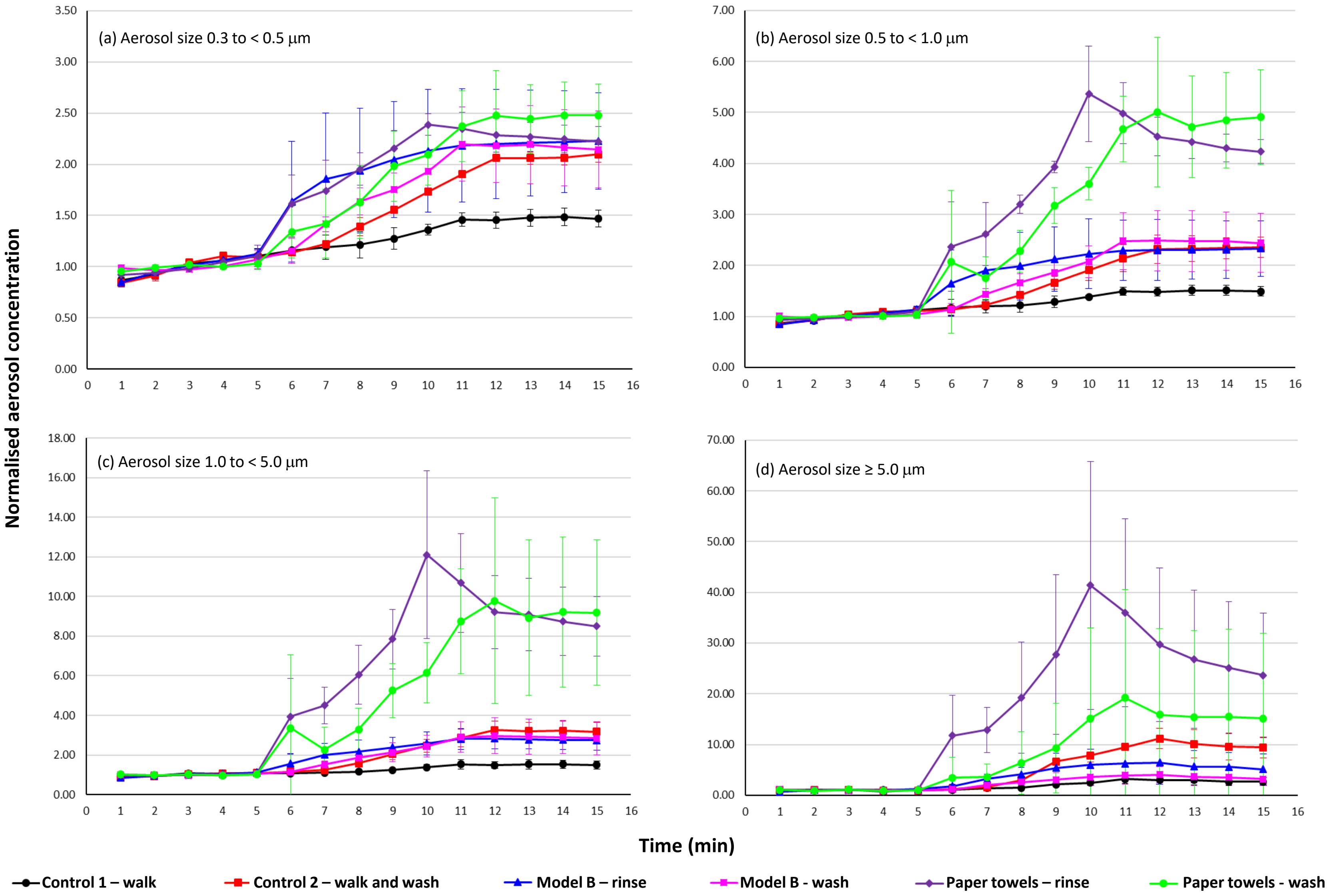

Figure S1.2. Representation of normalised data for model B for aerosol bins a) 0.3, b) 0.5, c) 1.0 and d) 5.0 for location 1 (side of hand dryer). Each curve represents (●) control 1 (walking only), (■) control 2 (walking and hand washing), and drying hands with jet dryer model B after (▲) rinsing and (■) washing, and drying with paper towels after (◆) rinsing and (●) washing. Vertical bars represent standard deviation of three experiments.

S1 – Normalised aerosol concentration with time

Location 1

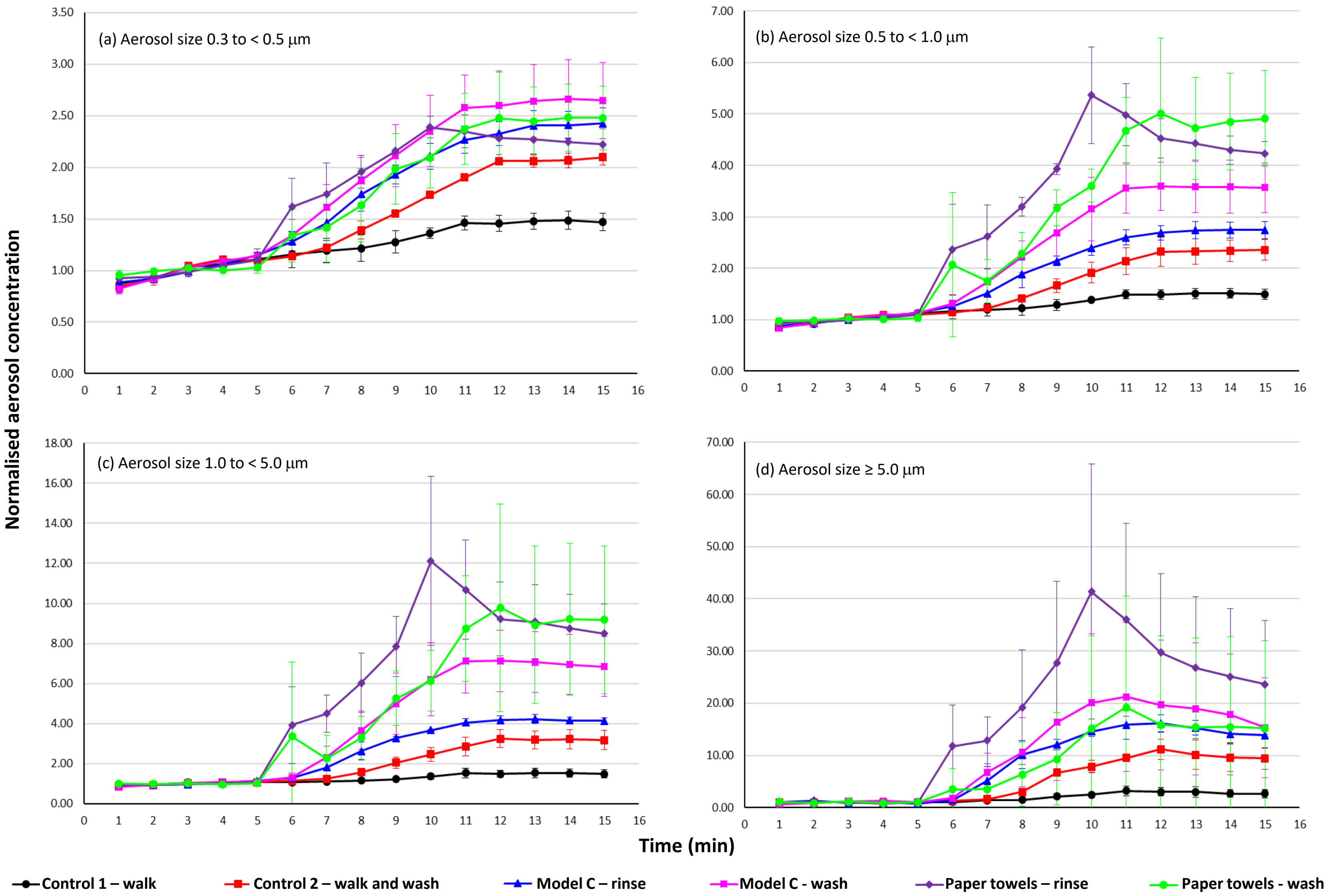

Figure S1.3. Representation of normalised data for model C for aerosol bins a) 0.3, b) 0.5, c) 1.0 and d) 5.0 for location 1 (side of hand dryer). Each curve represents (●) control 1 (walking only), (■) control 2 (walking and hand washing), and drying hands with jet dryer model C after (▲) rinsing and (■) washing, and drying with paper towels after (◆) rinsing and (●) washing. Vertical bars represent standard deviation of three experiments.

S1 – Normalised aerosol concentration with time

Location 1

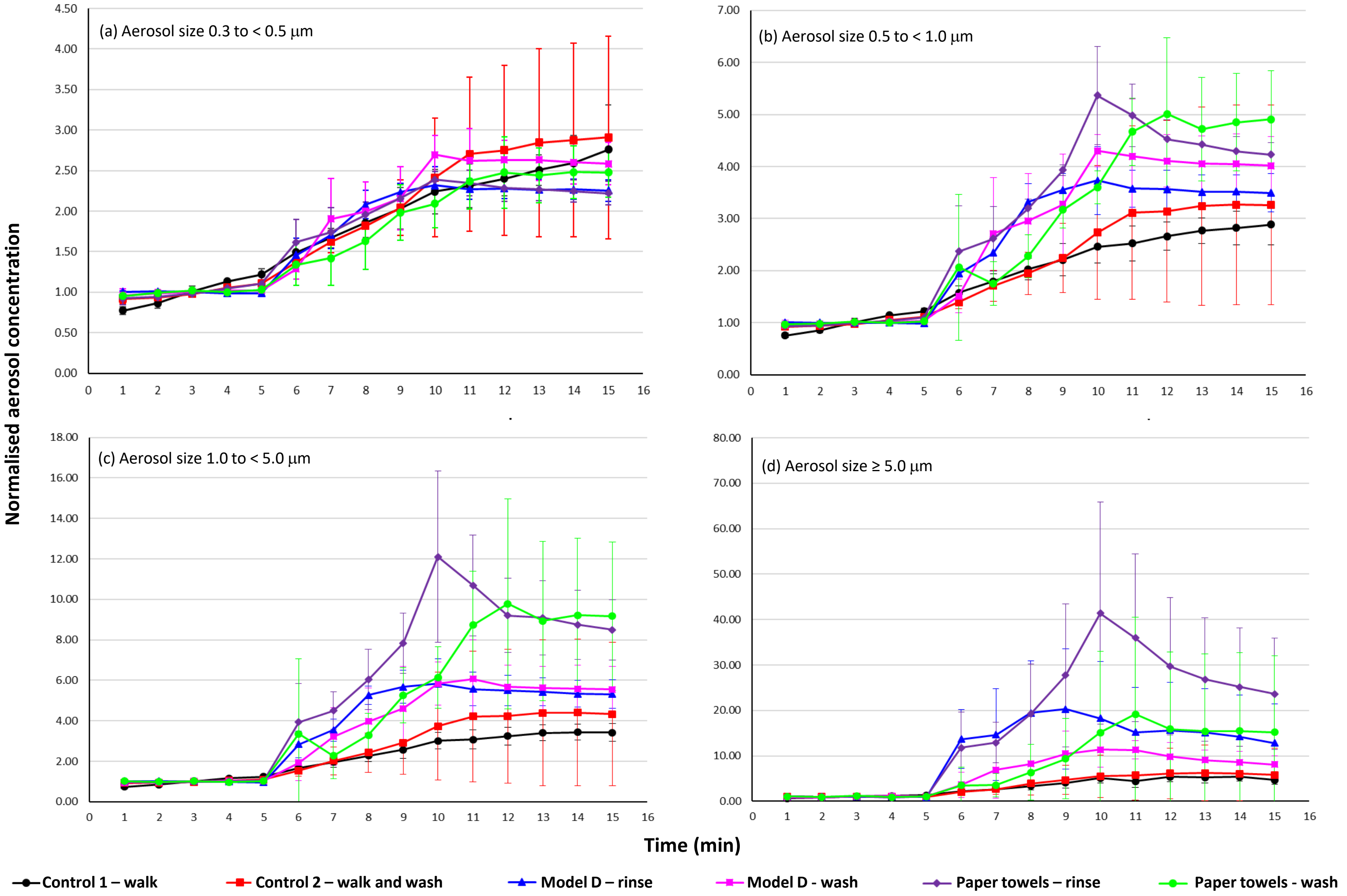

Figure S1.4. Representation of normalised data for model D for aerosol bins a) 0.3, b) 0.5, c) 1.0 and d) 5.0 for location 1 (side of hand dryer). Each curve represents (●) control 1 (walking only), (■) control 2 (walking and hand washing), and drying hands with jet dryer model D after (▲) rinsing and (■) washing, and drying with paper towels after (◆) rinsing and (●) washing. Vertical bars represent standard deviation of three experiments.

S1 – Normalised aerosol concentration with time

Location 2

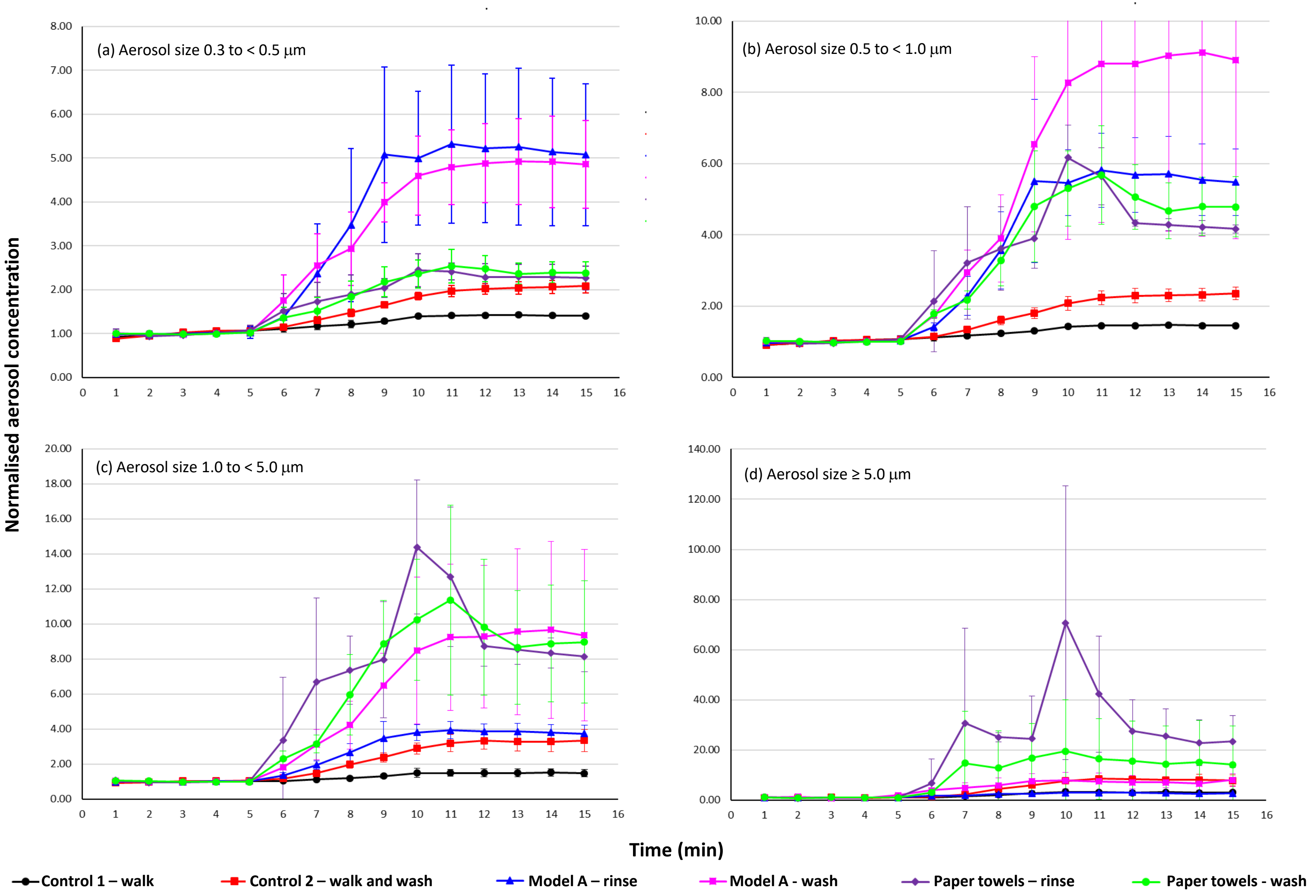

Figure S1.5. Representation of normalised data for model A for aerosol bins a) 0.3, b) 0.5, c) 1.0 and d) 5.0 for location 2 (near the hand dryer). Each curve represents (●) control 1 (walking only), (■) control 2 (walking and hand washing), and drying hands with jet dryer model A after (▲) rinsing and (■) washing, and drying with paper towels after (◆) rinsing and (●) washing. Vertical bars represent standard deviation of three experiments.

S1 – Normalised aerosol concentration with time

Location 2

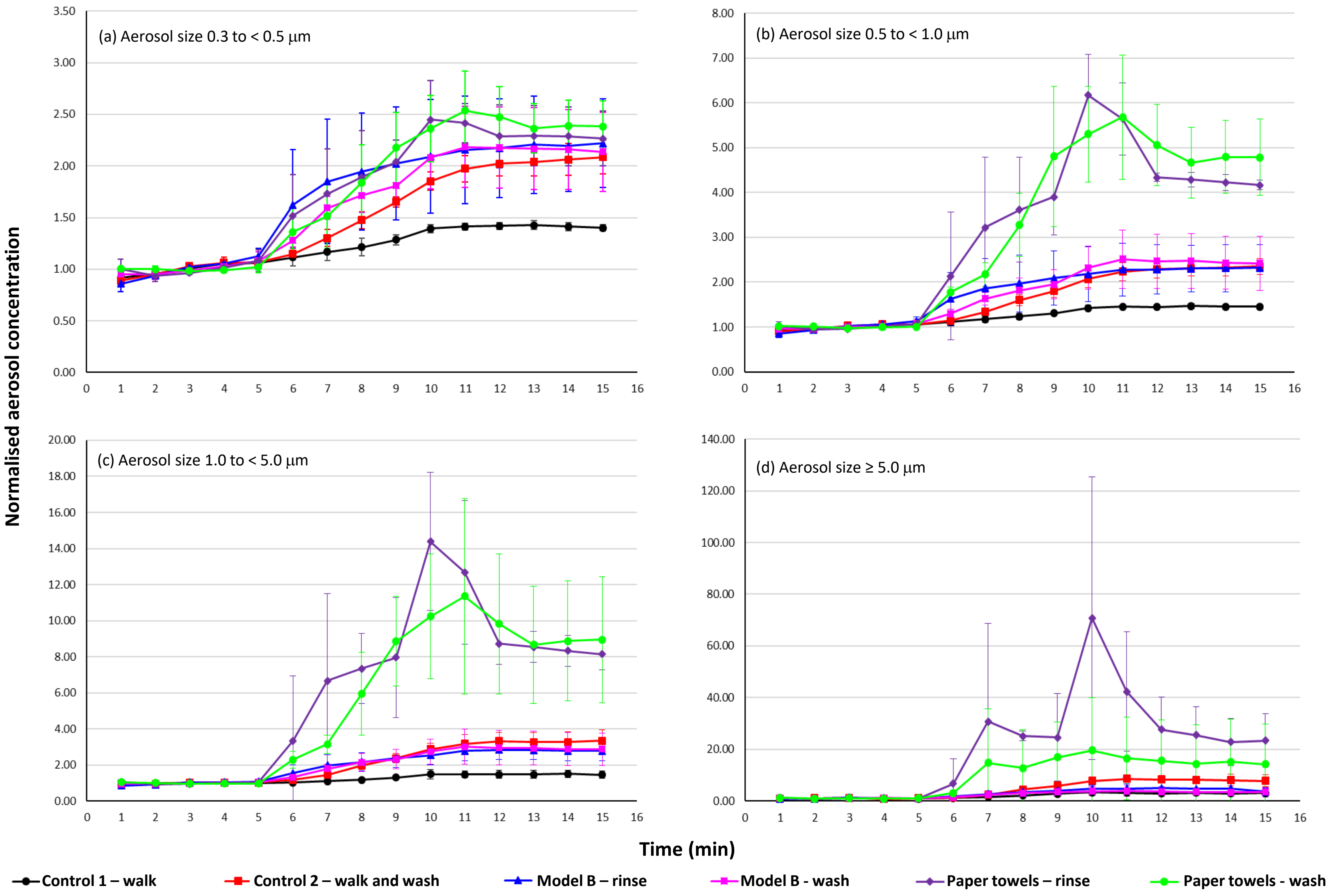

Figure S1.6. Representation of normalised data for model B for aerosol bins a) 0.3, b) 0.5, c) 1.0 and d) 5.0 for location 2 (near the hand dryer). Each curve represents (●) control 1 (walking only), (■) control 2 (walking and hand washing), and drying hands with jet dryer model B after (▲) rinsing and (■) washing, and drying with paper towels after (◆) rinsing and (●) washing. Vertical bars represent standard deviation of three experiments.

S1 – Normalised aerosol concentration with time

Location 2

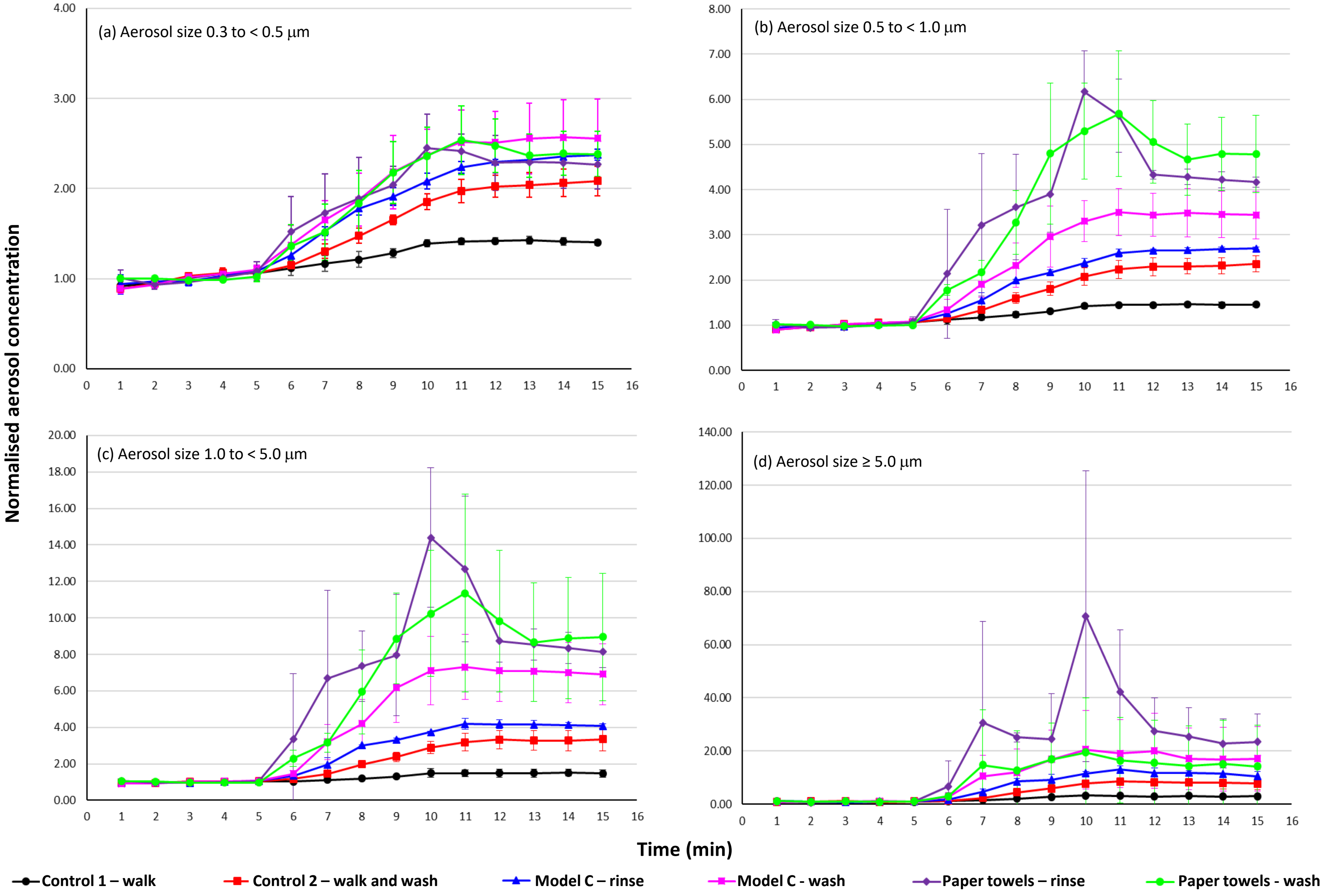

Figure S1.7. Representation of normalised data for model C for aerosol bins a) 0.3, b) 0.5, c) 1.0 and d) 5.0 for location 2 (near the hand dryer). Each curve represents (●) control 1 (walking only), (■) control 2 (walking and hand washing), and drying hands with jet dryer model C after (▲) rinsing and (■) washing, and drying with paper towels after (◆) rinsing and (●) washing. Vertical bars represent standard deviation of three experiments.

S1 – Normalised aerosol concentration with time

Location 3

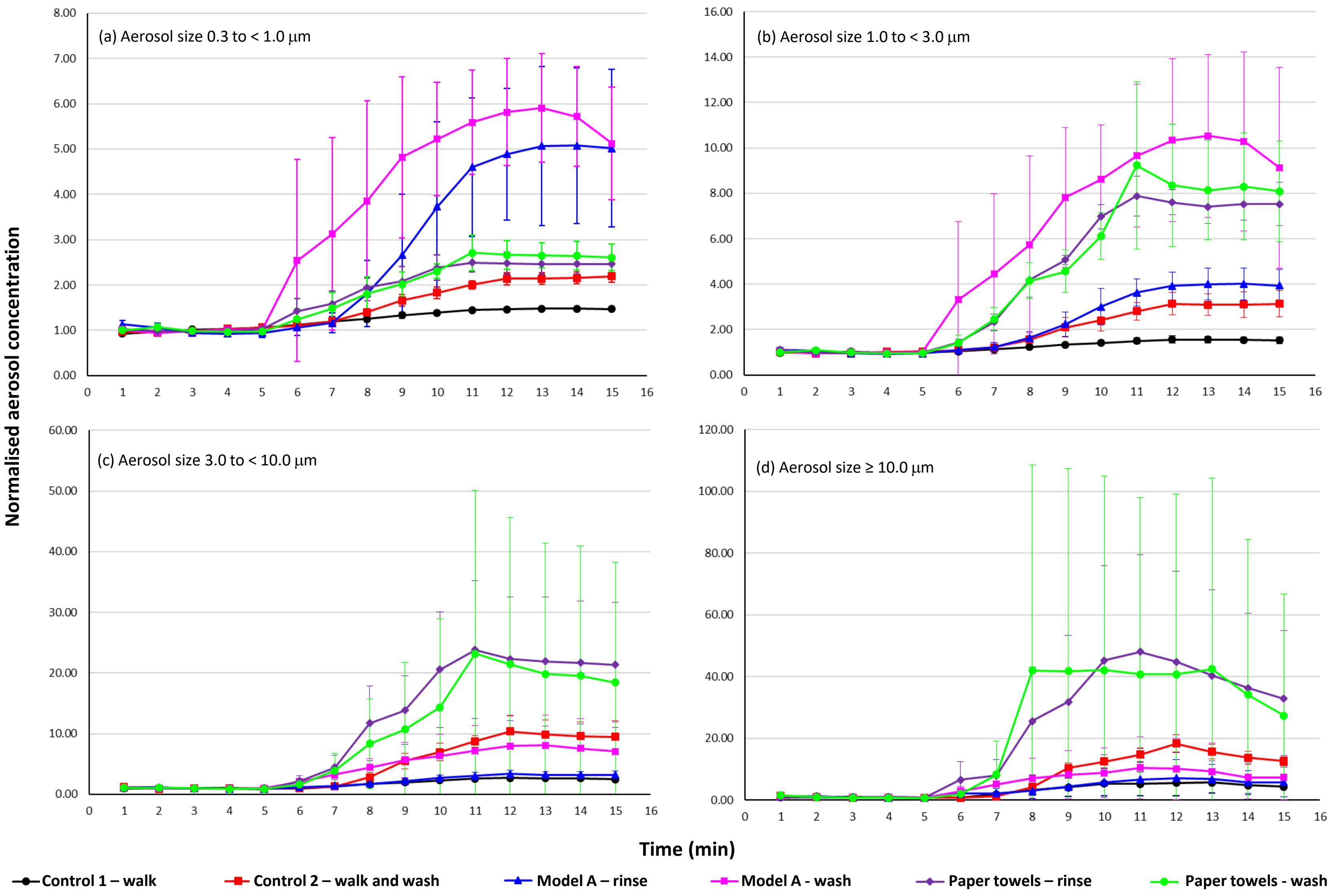

Figure S1.8. Representation of normalised data for model A for aerosol bins a) 0.3, b) 1.0, c) 3.0 and d) 10.0 for location 3 (opposite the hand dryer). Each curve represents (●) control 1 (walking only), (■) control 2 (walking and hand washing), and drying hands with jet dryer model A after (▲) rinsing and (■) washing, and drying with paper towels after (◆) rinsing and (●) washing. Vertical bars represent standard deviation of three experiments.

S1 – Normalised aerosol concentration with time

Location 3

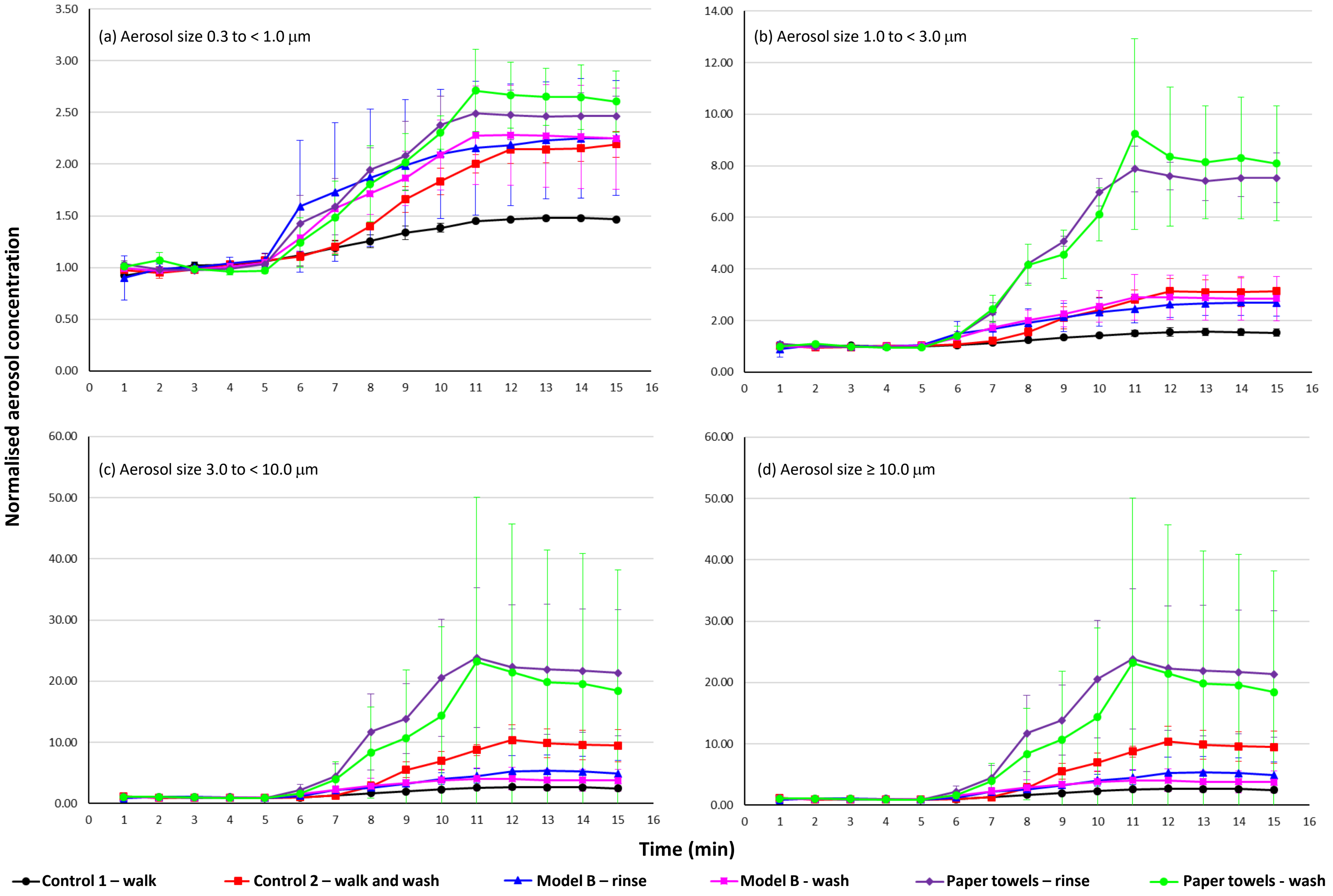

Figure S1.9. Representation of normalised data for model B for aerosol bins a) 0.3, b) 1.0, c) 3.0 and d) 10.0 for location 3 (opposite the hand dryer). Each curve represents (●) control 1 (walking only), (■) control 2 (walking and hand washing), and drying hands with jet dryer model B after (▲) rinsing and (■) washing, and drying with paper towels after (◆) rinsing and (●) washing. Vertical bars represent standard deviation of three experiments.

S1 – Normalised aerosol concentration with time

Location 3

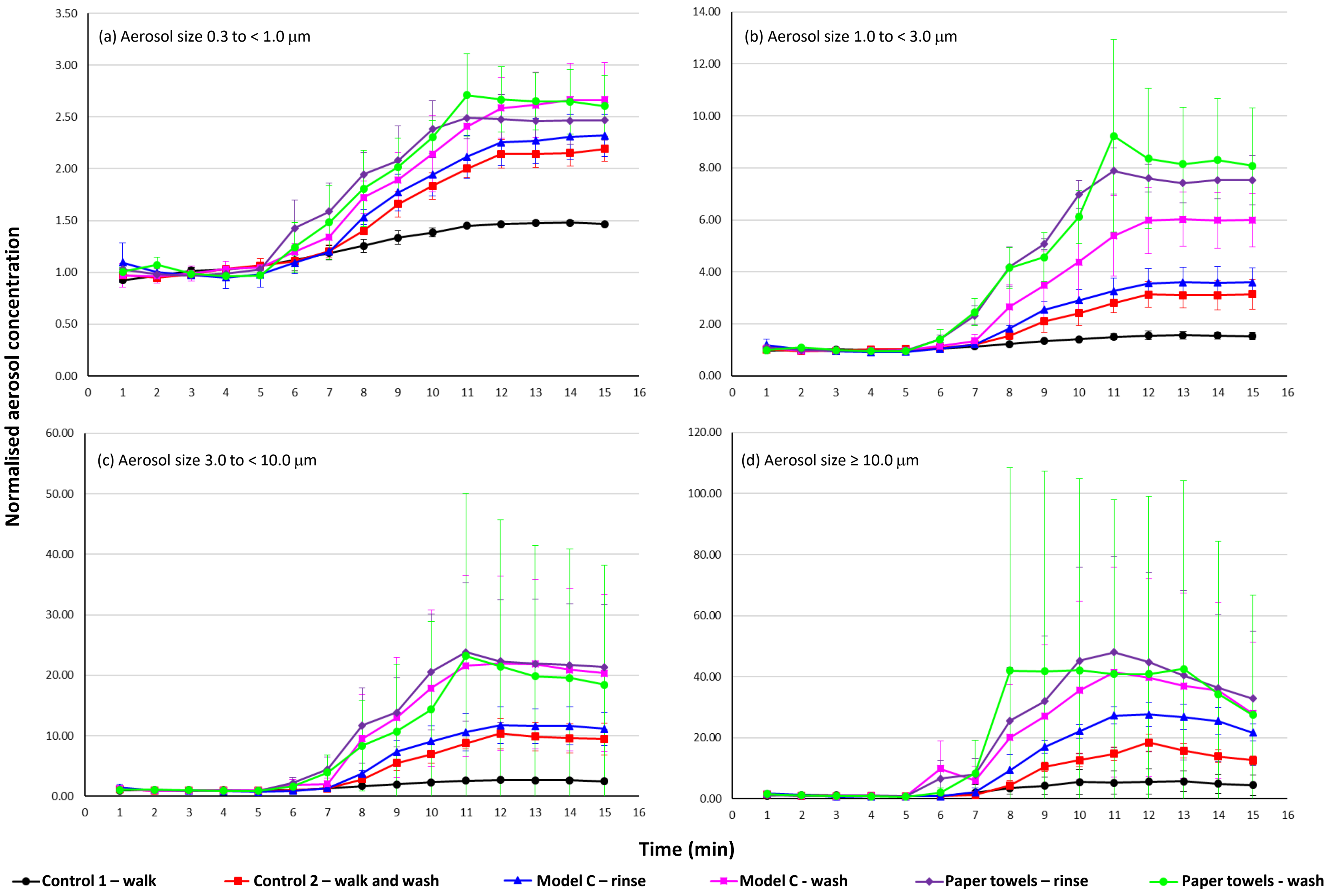

Figure S1.10. Representation of normalised data for model C for aerosol bins a) 0.3, b) 1.0, c) 3.0 and d) 10.0 for location 3 (opposite the hand dryer). Each curve represents (●) control 1 (walking only), (■) control 2 (walking and hand washing), and drying hands with jet dryer model C after (▲) rinsing and (■) washing, and drying with paper towels after (◆) rinsing and (●) washing. Vertical bars represent standard deviation of three experiments.

S1 – Normalised aerosol concentration with time

Location 3

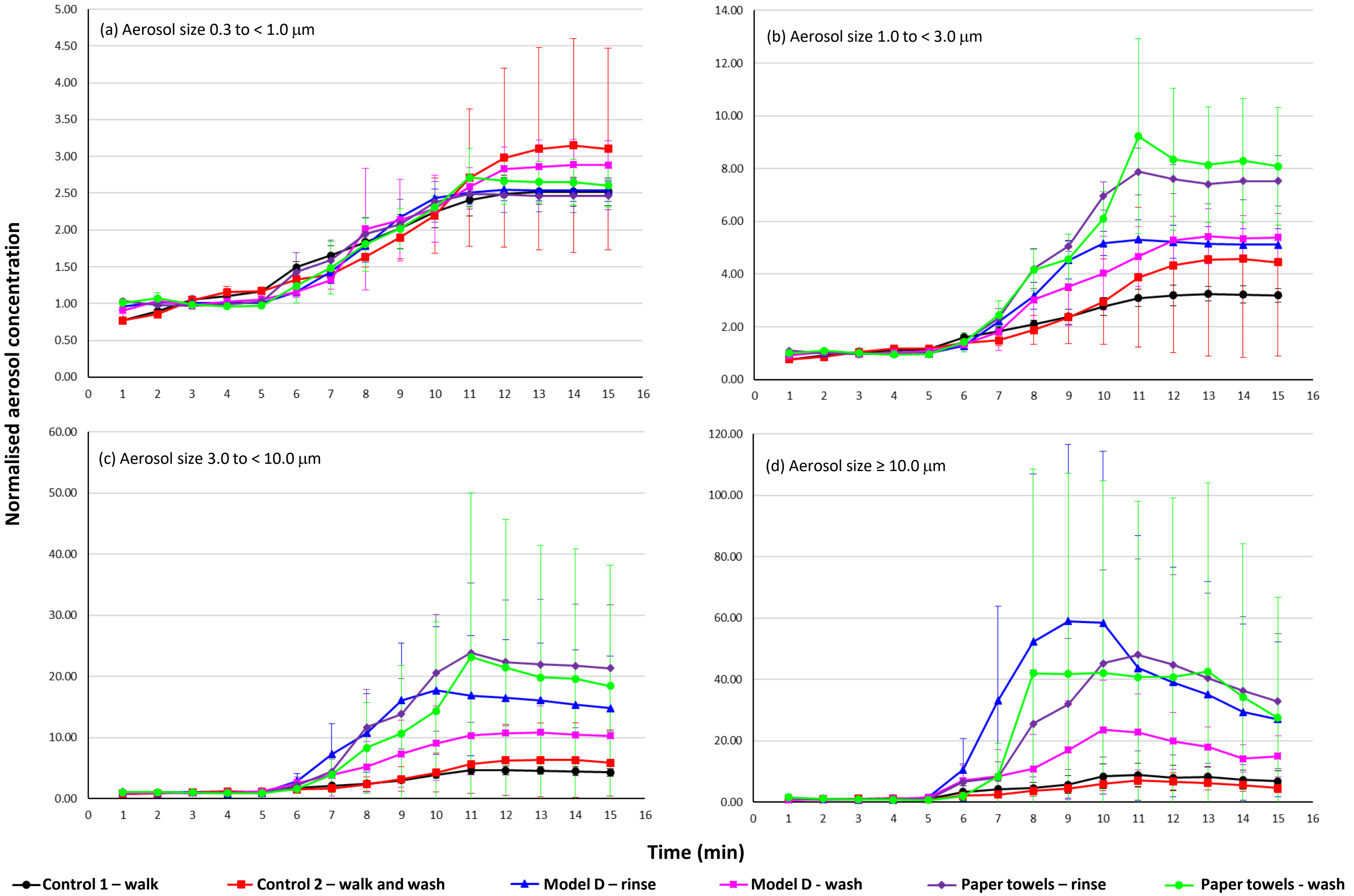

Figure S1.11. Representation of normalised data for model D for aerosol bins a) 0.3, b) 1.0, c) 3.0 and d) 10.0 for location 3 (opposite the hand dryer). Each curve represents (●) control 1 (walking only), (■) control 2 (walking and hand washing), and drying hands with jet dryer model D after (▲) rinsing and (■) washing, and drying with paper towels after (◆) rinsing and (●) washing. Vertical bars represent standard deviation of three experiments.

S2 – Average aerosol concentration

Location 1

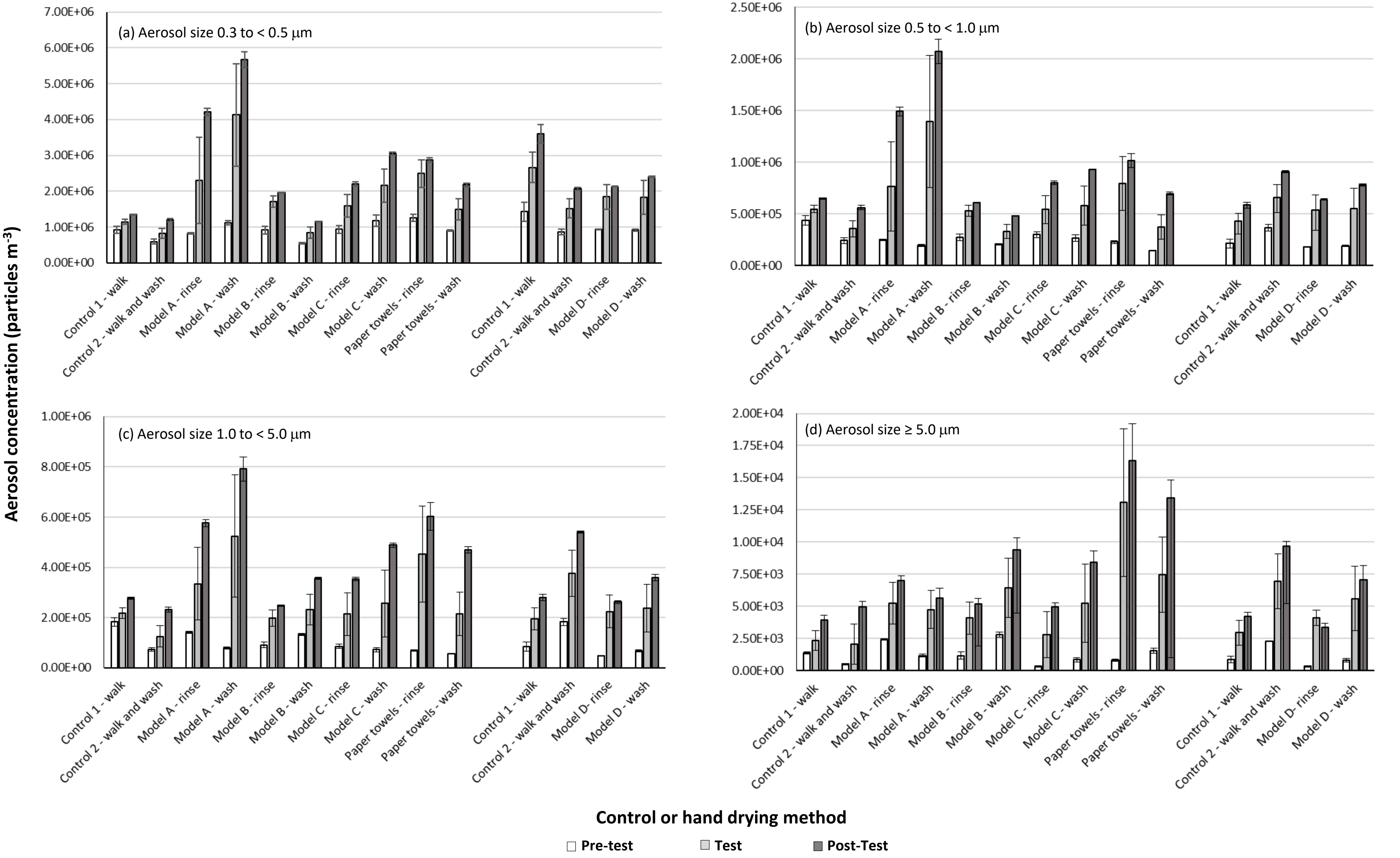

Figure S2.1. Representation of average of 5 time points for aerosols concentration for aerosol bins a) 0.3, b) 0.5, c) 1.0 and d) 5.0 for controls and hand drying methods for location 1 (side of hand dryer). Each bar represents: white bars – pre-test (before volunteers enter the chamber), light grey bars – test (volunteers in the chamber) and dark grey bars – test (after volunteers leave the chamber). Vertical bars represent standard deviation of three experiments.

S2 – Average aerosol concentration

Location 2

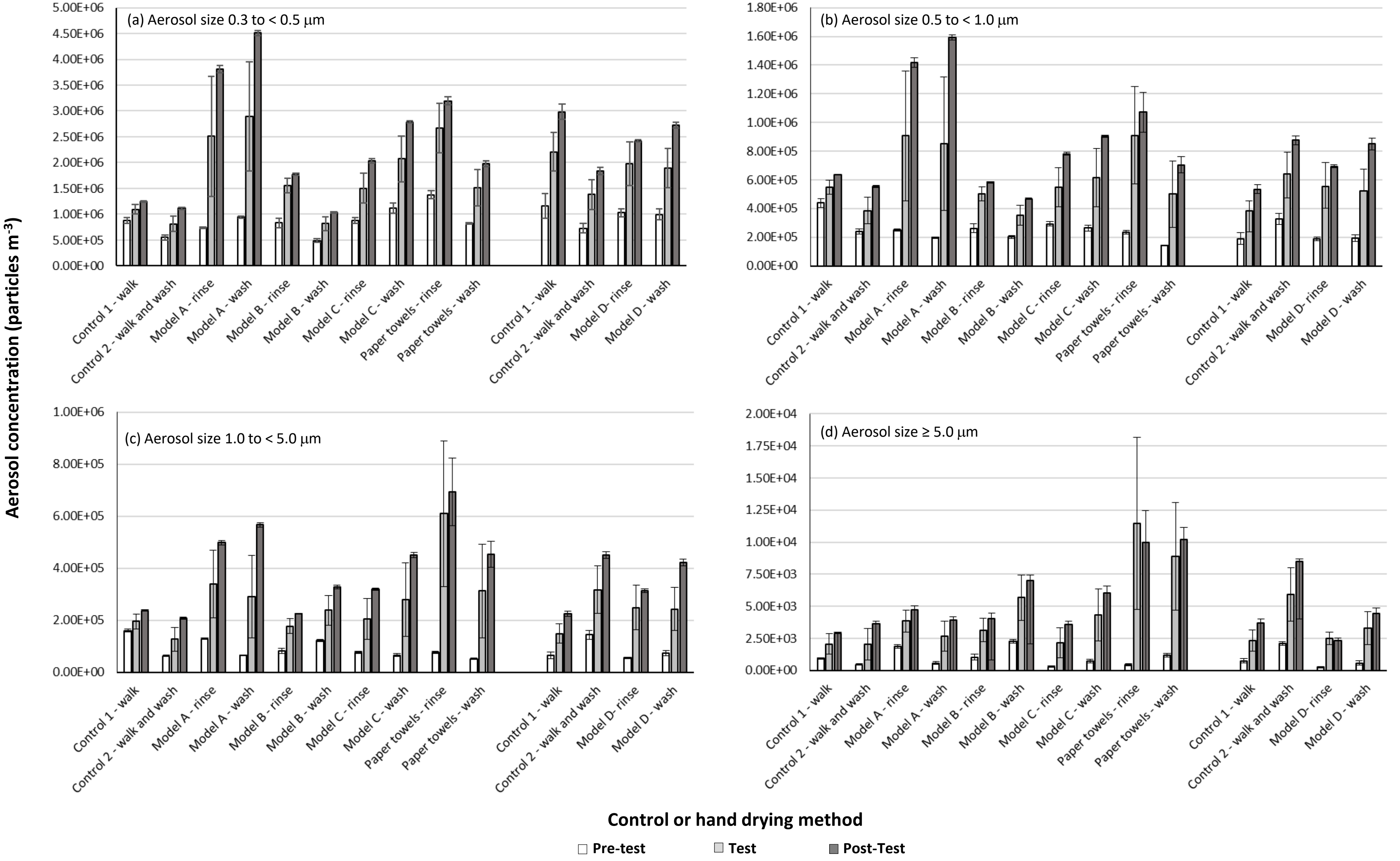

Figure S2.1. Representation of average of 5 time points for aerosols concentration for aerosol bins a) 0.3, b) 0.5, c) 1.0 and d) 5.0 for controls and hand drying methods for location 2 (near hand dryer). Each bar represents: white bars – pre-test (before volunteers enter the chamber), light grey bars – test (volunteers in the chamber) and dark grey bars – test (after volunteers leave the chamber). Vertical bars represent standard deviation of three experiments.

S2 – Average aerosol concentration

Location 3

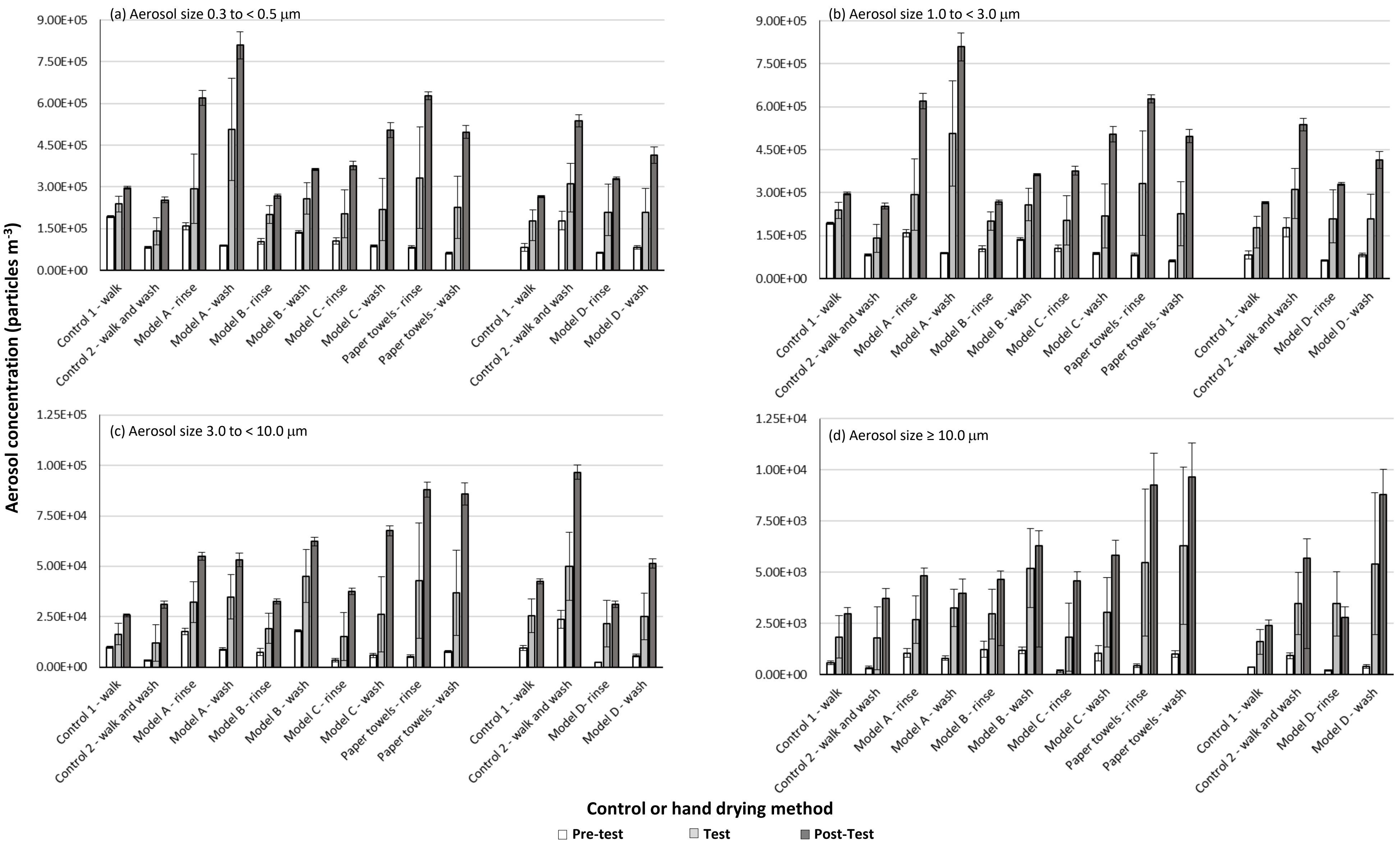

Figure S2.3. Representation of average of 5 time points for aerosols concentration for aerosol bins a) 0.3, b) 1.0, c) 3.0 and d) 10.0 for controls and hand drying methods for location 3 (opposite the hand dryer). Each bar represents: white bars – pre-test (before volunteers enter the chamber), light grey bars – test (volunteers in the chamber) and dark grey bars – test (after volunteers leave the chamber). Vertical bars represent standard deviation of three experiments.
